# Mediating Role of Resilience in the Relationship Between English Learners’ Motivation and Well-Being

**DOI:** 10.3389/fpsyg.2022.915456

**Published:** 2022-05-24

**Authors:** Fei Wang, Yanghong Liu

**Affiliations:** School of Foreign Languages, Xinyang College, Xinyang, China

**Keywords:** well-being, English learners, mediating role, motivation, resilience

## Abstract

Teaching seeks to enhance learners’ well-being as well as their educational motivation since both constructs cause advancement in the process of learning and they increase the level of success in the educational cycle. Well-being is a critical requirement inside the academic environment that is considered as the main dimension of individuals’ tendency in the topic of positive psychology (PP) research, which is crucial for learning. Besides, as a significant idea in language domain and in order to consider the relation between well-being and motivation, the other concept is arisen in PP, namely resilience that seems to be effective for learners as it deals with the capability to effectively manage difficulties in the past and present time in the learning process. As a result, this study considers the relationship between well-being and motivation; however, it also inspects the mediating role of resilience in this regard. Consequently, this review of literature holds suggestions for researchers, philosophers, and experts searching for better exploration and attention to the functions of resilience as a mediator in the relationship between learners’ motivation and well-being.

## Introduction

It is stated that personal variations have always been considered as fixed predictors of achievement in language learning (Dörnyei and Skehan, 2003). Language anxiety, motivation, aptitude, and attitudes are comparatively prevailing study agendas relating to second language learning (Dewaele and MacIntyre, 2014; Dörnyei and Ryan, 2015). For a long time, among which, motivation has been the core of human success and attainment, both in individual and scholastic life (Gopalan et al., 2018). As the principal and prominence of human manner, motivation has additionally been understood as one of the crucial personal variations able to assist learners’ educational achievement that is largely examined within the academic setting in different dimensions and perspectives, and also its function on learners’ fulfillment and education is assured (Al-Hoorie, 2017). In addition, recently, the effective role of motivation has been questioned in previous studies that affect the efficiency of language education (Dörnyei and Ushioda, 2013) which is the primary cause why people select to do a task, the time they would spend on it, and how strongly they are decided to follow it (Dörnyei and Skehan, 2003). The findings of studies regarding the useful role of motivation in language learning show that one can firmly assert that there is a strong correlation between the assumed power of motivation and the degree of second language success which makes the significant correlation probably highly significant (Saville-Troike, 2006). In a nutshell, the main factor leading to learning is motivation and, subsequently, has an effective role that enables students to continue the extensive procedure of learning a foreign language (Shiri, 2015).

Alternatively, besides motivation, in the past 10 years, with the advent of a rather new branch of psychology as positive psychology (PP), research have significantly highlighted the powers, property, and abilities of people that cause final performance, through which, there has been a change in the focus from sickness to well-being (Seligman, 2011). Undoubtedly, the focus on students’ well-being in the school setting is on the rise and as well-being is related to extensive educational and mental results, that is, lower stress during exams, lower symptoms of depression and exhaustion (Steinmayr et al., 2016), in addition to better rates of success, positive behavioral manner, and adaptation to change (Putwain et al., 2019). Indeed, one of the key concepts investigated within the PP area is well-being because it pertains to a vast range of academic and psychological outcomes encompassing lower stress, fatigue, and burnout besides higher rates of achievement, adaptability to changes, and constructive behavioral conduct (Derakhshan et al., 2021). Schools should turn into constructive organizations, with a remarkable emphasis both on learners’ educational functioning as well as their features and well-being (Peterson, 2009). Therefore, PP pursues to enhance the well-being of participators, as well as increase their achievement, meaning, performance, and flexibility in all fields, especially education (Prior, 2019). The PP aims at expanding positive emotions, fostering caring and useful relations, and setting up a feeling of persistence in living, rather than managing difficulties (Seligman, 2011). Currently, scholars mostly agree that well-being is essential for learners for optimal performance in school and protection from possible hazardous stressing factors (Forrest et al., 2013). Currently, PP is used in the area of learning language and education and mastering motivation. The educational attention as a secondary component of PP is starting to pay attention to well-being and resilience (Oxford, 2016; Wang et al., 2021).

Having motivated learners is good, but educational development is vulnerable to being unaccomplished when facing problems, pressure, or stress within the school environment without resilience, i.e., motivation is not the only cause of learners’ achievement (Martin, 2002). As an emerging field of study, the resilience concept got into the field of learning a second language. According to Martin (2002), such an emerging idea in academics means students’ capability to efficiently handle failing in mastering, studying pressures, and stress. Resilience is also an important factor in learners managing problems in learning conditions and continuing lengthy English mastering periods. However, the resilience notion was firstly used in the area of learning English. Few studies exist, both foreign and domestic, on learning English and resilience.

As a mental dimension of students, resilience has been at the center of attention in various domains, including progressive and academic psychology. The language educational cycle can be typically meticulous and can take time, and students could face different challenges when studying a foreign language. Resilience is prone to function as a personal distinction element that assists foreign language students with not surrendering so quickly and with conquering serious challenges in the long-term L2 educational cycle (Smith, 2006). Resilience is an essential personal feature in improving well-being which is related to an extensive scope of outcomes associated with fitness. Based on the studies regarding resilience, there is a negative relationship between resilience and perceived tension, anxiety, negative affect, depression, and bodily symptoms. However, it has a positive relationship with positive effects either in non-clinical or clinical samples (Smith et al., 2008). When encountering negative results, resilience becomes a defensive element and consequently assists people in keeping their mental and physical well-being (Connor and Davidson, 2003). Resilience has an important function in the educational domain as a motivational-affective factor in the sense that it is both a trigger for the attainment of educational and individual objectives and offers sufficient methods to cope with difficult circumstances of anxiety and worry that emerge in the college setting (González-Torres and Artuch-Garde, 2014; Cassidy, 2015). Educational resilience is the learners’ skill to cope successfully with failures, demanding situations, and pressure within the college setting over time (Fallon, 2010). It can increase the chance of achievement in school and the various domains of life despite environmental difficulties caused by early attributes, circumstances, and encounters. Moreover, Khalaf (2014) has characterized educational flexibility as one of the predictors of adaptation with the failures of college life and as one of the most powerful indicators of classroom engagement, taking pleasure in learning, and overall self-confidence.

Although the significance and position of useful variables such as persistence, efficiency, well-being, dedication, motivation, and resilience are proven in earlier inspections predicting the essential instructional outcomes (Ellahi Shirvan et al., 2021; Sulis and Philp, 2021), a dearth of studies can be felt regarding how they can work as mediators since based on literature review, they can act help learners in the learning progress. For example, being resilient help learners to cope with stress in face of difficulties that invoke their motivation. Indeed, nowadays, learners encounter lots of stress during language learning procedures and a number of them cannot properly manage their stress, and instead, this issue ends in the decline of well-being and improvement of demotivation (Ang and Huan, 2006), in this way, it can be concluded that being persistent to problems or demanding situations, and being capable of using a wide variety of tactics involving problem-solving also are vital to foreign language students’ achievement (Beachboard et al., 2011), while motivation and well-being are critical in the foreign language learning process. Nevertheless, only a handful of research is found that concentrate, particularly on constructive mental factors in learning a language, namely, resilience (Ushioda, 2008) and few studies could be found that focus specifically on positive psychological elements such as resilience in language learning (Yun et al., 2018). Regarding the above-mentioned statements confirming the important role of resilience within the process of learning and because of the relationship of both concepts, motivation, and well-being, the current review makes an effort to inspect the relationship between motivation and well-being and the mediator role of resilience in this realm.

## Key Concepts

### Motivation

Relying on the internalization level, motivations are categorized for people’s manner in self-determination theory (SDT; Deci and Ryan, 2000). They presented the continuum of SDT according to the internalization level. Amotivation is one the left end of the continuum, i.e., lacking the intention to perform. Amotivation refers to the lack of tendency to participate in mastering because of a student’s disability or indolence in a particular state (Deci and Ryan, 2016). Intrinsic motivation lies at the right side of the continuum, i.e., performing an activity due to desire and inner happiness. Students with intrinsic motivation are inclined to get involved in activities due to specific acts, desires, delight, and learning interests. On the contrary, students with external motivation conduct specific activities for the sake of prizes with characteristics, namely, applaud good scores, and higher status. Manners with extrinsic motivation, characterized by four kinds of regulation are located across the self-dedication amotivation—intrinsic motivation continuum. External motivation refers to motivation originating from expecting rewards presented extrinsically (Deci and Ryan, 2016). Based on this taxonomy, motivation to study can be boosted through external or inherent reasons. Students who learn for external reasons do so to receive benefits (e.g., doing well on an exam) or to evade penalizing (e.g., doing poorly on an exam). Contrastingly, inherently motivated students are inspired to learn because of the fulfillment they attain from studying. Such students are grateful for the unconventionality and demands associated with studying a language and go after chances to increase their linguistic capabilities to develop themselves as second language users (Dincer and Yesilyurt, 2017). Out of the two, inherently motivated students are more prone to persevere in their language research with time and even when facing impediments since they take pleasure in being involved in studying the language (MacIntyre et al., 2019). As for EFL educators, promoting inherent motivation in their students can be more beneficial for long-run achievement in language learning.

Based on the theory of self-determination, human beings can be eager and psychologically well-adapted in case of having several opportunities to satisfy these three fundamental demands (Deci and Ryan, 2016). Motivation addresses the wisdom of those who make unique selections, interact in activities and insist on searching for them which also manages the level of severe private engagement in language learning (Ushioda, 2008). Motivation provides the main drive to begin learning a language and after a while the excuse for pursuing the expanded and boring learning procedure (Dornyei, 2005). Dornyei believed that motivation contributes to the whole elements related to gaining knowledge of a second language. Also, it is stated that motivation pertains to the motive of people to make a specific decision, to engage in a task, and to persist in following it and it adjusts the greatness of strength, personal engagement in learning a second language (Ushioda, 2008).

### Resilience

Luthar et al. (2000) presented the resilience idea to the literature and described it as a dynamic procedure including people’s positive compatibility when encountered with considerable hardships. The construct of resilience pertains to psychology and the human improvement theory which is described as a running procedure including positive compatibility when facing severe difficulty, or people’s ability to recover from hardships and adjust to their setting (Wagnild and Collins, 2009). However, several scholars defined resilience as a result decided by an individual’s precise manner despite the risk or as a dynamic procedure wherein optimistic compatibility emerges in a difficulty including the interplay of hazard and securing elements ranging from the person to the community (Olsson et al., 2003). As a significant factor in optimistic psychology, resilience means an individual’s flexibility to address diverse problems and difficulties in life and to recover from difficulties and depression (Dent and Cameron, 2003). Based on the findings, resilience has a crucial role in lowering the anxiety and tension about mastering a new language, thus, enhancing the experience of language learning (Chaffee et al., 2014). In addition, Ryff and Singer (2003) argue that resilient people can in general keep their physical and mental health and have the potential to restore faster when facing situations full of pressure because they adapt high degrees of successful fulfillment and motivation despite numerous stressing situations.

Individuals who are resilient persevere more when encountering difficulties; strive harder during jeopardizing situations; cope successfully with anxiety and distress; have a greater capability of reacting to life troublesomeness, and deal with daily challenges better (Smith, 2006). Consistent with Martin and Marsh (2009), academically resilient students can maintain higher levels of achievement, encouragement, and presentation in spite of the presence of stressful events and moods that makes them vulnerable to failure in school and, finally, dropping out of school. The literature review shows that the resilience notion is employed in a vast series of various spheres, to state the system persistence, a structure or a public directed to the disorder or interrupting variations prompted by conditions of human beings, society, or ecology (Tudo and Spray, 2017).

### Well-Being

When the PP emerged, scholars concentrated on both improving negative moods as well as increasing positive moods, personal powers, and their association with intellectual peace, well-being, and social success. Positive psychology highlights the significance of recognizing and comprehending elements related to personal well-being, mental well-being, and prosperity (Seligman and Csikszentmihalyi, 2014). Positive psychology highlights the prominence of classifying and understanding issues related to subjective well-being, psychological well-being, and thriving. The objective of positive psychology is to enhance personal well-being and enjoyment. Positive psychologists have the objective of improving mental functioning and personal achievement (Seligman, 2018). As a component of PP, well-being is characterized as a positive and lasting quality that enables individuals and societies to flourish and grow.

There are two research paradigms related to well-being that is characterized under two perspectives and they arise from different Greek philosophies: hedonism and eudemonism (Ryan and Deci, 2017). Hedonic well-being alludes to optimizing enjoyment and satisfaction while reducing hurt and distress (Diener et al., 2003). The organismic valuing cycle and humanistic psychology’s recognition of the good life possibly mirror the eudemonic method to the well-being more, which highlights the significance of development towards greatness, growth, significance, and reality (Huta and Waterman, 2014). Well-being is an idea with multiple aspects of positive experience, and performance and is typically taken from two large methods (Deci and Ryan, 2000). One of them is mental well-being or joy, which is based on hedonism, meaning the experience of satisfaction and pain avoidance described as what people reflect and the way they conceive their living at the time of cognitive and emotional recognition regarding their entity (Seligman and Csikszentmihalyi, 2014). Mental well-being refers to a trilateral model made of three varieties, but related factors, namely, the existence of repeated positive affections, the rare occurrence of negative affections, and life satisfaction (Diener et al., 2003). Psychological well-being (PWB) is a central notion inside the eudemonic paradigm (Ryff and Singer, 2008); great levels of PWB are attained when one obtains the internal assets for sustaining well-being. In particular, these assets include a sense of self-worth, constructive connections with others, and a sense of mastering one’s achievement (Ryff and Singer, 2008).

Seligman (2018) proposed that well-being has various elements, and numerous measurable components exist that assist in building the well-being conceptual context called the hypothesis of PREMA with five topics, including positive affections, relations, engaging, meaningfulness, and attainment. Positive feelings involve fulfillment, optimism, and well-being, which are regarded as components of the hedonic continuum of emotional states that function as indicators of achievement because they can help people with flourishing that can be taught and improved (Fredrickson, 2001). Engagement is regularly alluded to as a form of flow or deep involvement that is primarily intended to be motivating throughout the achievement of a task (Derakhshan, 2021). Goal setting, observing, and attainment heighten well-being throughout an entire life course (Heckhausen et al., 2010). Positive relations refer to a sense of being socially accepted, noticed, and strengthened, and taking pleasure in one’s social relationship. Social help is connected to positive outcomes of psychological and physical well-being as well as to general well-being (Greenier et al., 2021). Meaning is the concept that a person’s life has persistence and a means in the course of life that is associated with being linked to something bigger and with constructive feelings in different age ranges (Yang, 2021). Attainment is commonly linked to goal setting, growth, and possessing the ability to achieve, thus, striving for well-being (Fredrickson, 2001).

## Implications and Future Directions

The considerable results in this review increased the developing body of studies in the PP area. Well-being and motivation significantly affect investigating and recognizing elements which result in the improvement of one’s eagerness to address difficulties in the process of language learning. Subsequently, the present findings supported the prior ones suggesting that resilience can be a protective issue that complements students’ achievement. This issue suggests that students who have high degrees of resilience will more likely experience higher joy, performance, and life satisfaction. The present study considerably contributes to the academic policy-makers, school heads, and teachers in several different ways. When looking for methods of enhancing the motivation and well-being of learners, it is proved to be useful for school heads to build resilience within the academic system. The best significant help offered by the current research is that it expands the current literature since it offers a way that could moderately enlighten why educational resilience helps language students. As an instance, it improves learners’ motivation for gaining knowledge of the English language (Martin and Marsh, 2009). In addition, educators must offer learners chances to be motivated sufficiently in the class when facing difficulties. Therefore, they should help their students in the educational cycle and offer them strategies that could motivate them to be more involved in the class that brings about more motivation and well-being that both consequently lead to their success and achievement. Through making use of some teaching methods that particularly construct learners’ resilience, educators can also improve learners’ motivation and well-being. Therefore, before students’ exhaust due to educational tension or different issues, workshops can be included in the curriculum of learners to provide them with firmer resilience and constructive cognitions. Furthermore, to foster constructive perspectives regarding the self, the universe, and the future, households, teachers, and consultants need to instruct the resilient spirit among learners in their socialization and training. With such constructive cognitions kept in mind, learners can have a higher ability to manage their difficult situations and enjoy well-being when facing challenging situations or hardships. All the beneficiaries in the academics, particularly educators and educator trainers can use the outcomes of the present study. Getting aware of students’ resilience and its effect on learners’ motivation can assist and encourage the educator trainers to consider an important issue which will result in getting higher academic results. Finally, including some preliminary courses allocated to growing and promoting such vital mental characteristics in students can be deemed as useful suggestions that are believed to help strengthen the academic efforts.

In addition, Waxman et al. (2012) also found that the language students with high and medium resilience perceived greater competition within the class. Moreover, Kim et al. (2018) proved the role of resilience as an obvious educational purpose that can stimulate learners’ lack of motivation for learning English. Indeed, learners with resilience indicate great motivation, problem-solving capabilities, social skills, and a tendency towards the target. Moreover, studies have shown that resilience significantly and positively as well as negatively affects the motivation and lack of motivation of students, respectively (Shin and Kim, 2017; Kim, 2019). Resilience acts as a mediator between well-being and motivation, relating to a person’s capability or considering difficulties as chances for developing intellect (Zeng et al., 2016), and resilience ought to be at the core of the attention of management since it is a crucial structure for comprehending how people deal with anxiety and sustain their degree of well-being. In order to enhance the motivated manner, improving resilience would be useful; indeed, whilst students recognize the second language learning advantages and connect language learning with their lives, they will have greater satisfaction with the process of language learning, which results in an increased level of motivation. Also, it can be beneficial to assist students to admit the unavoidable issues encountered during the language learning according to practical concepts. Students with great educational resilience have a tendency to work according to true futuristic programs and undertake accountability for their moves. As a result, students with great educational resilience are inclined to be superior in terms of cognition and academics compared to those students lacking educational resilience, and they keep their educational motivation (Rouse, 2001).

Therefore, through some types of tasks, namely, programs for emotion regulation, classes associated with goal scheduling, and help-seeking manners, schools should try to expand the improvement of learners’ resilience. As an important mediator in the motivation-resilience relationship, well-being suggests that satisfaction with life could enhance one’s perception of self-abilities and, as a result, lead to more resilience. Undoubtedly, well-being pertains to the cognitive belief regarding satisfaction with life; thus, enhancing people’s well-being ends in higher motivation and guarantees a resilient individual in the case of facing difficulties. A pleased person or those with greater well-being constantly think positively, and they are glad, and they are less influenced by stress. Well-being may lead to great sensation, improves resilience and as a result, decreases the effect of tension which will enhance motivation (Soave, 2014). Similarly, the faculty members could provide classes and seminars that consider the hypothetical and empirical dimensions of resilience for learners to promote their capacities in this domain. Moreover, more studies with various designs could be carried out to include data related to the factors of the current study like motivation and well-being to offer an inclusive insight into the problem. In further research, implementing some types of interferences is proposed to clarify the problem in a language setting.

Because resilience is a considerable mediator in the association of well-being and motivation, it can be suggested that research in the future employ experimental techniques to repeat those results to obtain a greater general comprehension of those associations. In this study, the findings indicated help for emerging plans for learners that concentrate on fostering resilience to enhance their capability to successfully control the complicated difficulties in the education process. More studies might be carried out to scrutinize other elements of resilience such as optimism in terms of its association to motivation since it is considered as a basis of motivation as optimist learners do not relinquish in the face of unacceptable situations.

## Ethics Statement

The studies involving human participants were reviewed and approved by Xinyang College Academic Ethics Committee. The patients/participants provided their written informed consent to participate in this study.

## Author Contributions

FW and YL have made a direct and intellectual contribution to the work, and got it ready for its publication. All authors contributed to the article and approved the submitted version.

## Conflict of Interest

The authors declare that the research was conducted in the absence of any commercial or financial relationships that could be construed as a potential conflict of interest.

## Publisher’s Note

All claims expressed in this article are solely those of the authors and do not necessarily represent those of their affiliated organizations, or those of the publisher, the editors and the reviewers. Any product that may be evaluated in this article, or claim that may be made by its manufacturer, is not guaranteed or endorsed by the publisher.
